# Investigating the Effect of Chain Connectivity on the Folding of a Beta-Sheet Protein On and Off the Ribosome

**DOI:** 10.1016/j.jmb.2018.10.011

**Published:** 2018-12-07

**Authors:** Andrew P. Marsden, Jeffrey J. Hollins, Charles O’Neill, Pavel Ryzhov, Sally Higson, Carolina A.T.F. Mendonça, Tristan O. Kwan, Lee Gyan Kwa, Annette Steward, Jane Clarke

**Affiliations:** Department of Chemistry, University of Cambridge, Lensfield Road, Cambridge, CB2 1EW, UK

**Keywords:** protein folding, folding on the ribosome, SecM, immunoglobulin-like, Phi value, GdmCl, guanidinium chloride, CPs, circular permutants, AP assay, arrest-peptide-based force-measurement assay

## Abstract

Determining the relationship between protein folding pathways on and off the ribosome remains an important area of investigation in biology. Studies on isolated domains have shown that alteration of the separation of residues in a polypeptide chain, while maintaining their spatial contacts, may affect protein stability and folding pathway. Due to the vectorial emergence of the polypeptide chain from the ribosome, chain connectivity may have an important influence upon cotranslational folding. Using MATH, an all β-sandwich domain, we investigate whether the connectivity of residues and secondary structure elements is a key determinant of when cotranslational folding can occur on the ribosome. From Φ-value analysis, we show that the most structured region of the transition state for folding in MATH includes the N and C terminal strands, which are located adjacent to each other in the structure. However, arrest peptide force-profile assays show that wild-type MATH is able to fold cotranslationally, while some C-terminal residues remain sequestered in the ribosome, even when destabilized by 2–3 kcal mol^−1^. We show that, while this pattern of Φ-values is retained in two circular permutants in our studies of the isolated domains, one of these permutants can fold only when fully emerged from the ribosome. We propose that in the case of MATH, onset of cotranslational folding is determined by the ability to form a sufficiently stable folding nucleus involving both β-sheets, rather than by the location of the terminal strands in the ribosome tunnel.

## Highlights

• Adjacent N and C terminal strands are most structured region in the transition state.
• Two circular permutants retain the same folding pathway as wild-type MATH.
• On the ribosome, early emergence of terminal strands does not promote earlier folding.
• Formation of both β-sheets is energetically critical for folding on the ribosome.
• Folding pathway minimizes formation of partly structured states prone to mis-folding.

## Graphical Abstract

**Figure:**
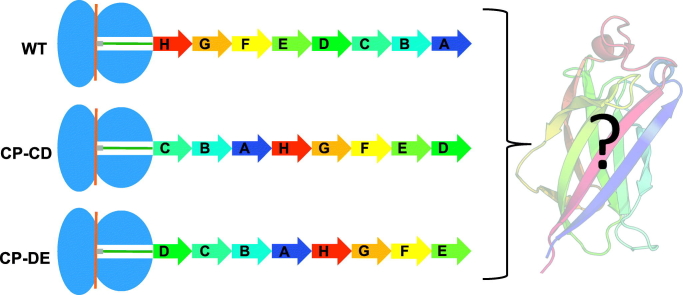

## Introduction

The relationship between the folding of domains in isolation and cotranslational folding on the ribosome has been investigated in recent years using a range of methods [1], [2] including studies that have compared *in vitro* folding with folding *in vivo* [3], [4], [5], [6], [7], [8], [9], [10], [11], [12]. Such studies have shown that elements of secondary and tertiary structure can form, and small peptides can fold, in the ribosome tunnel [13], [14], [15], [16] and that entire protein domains can fold in the exit vestibule of the ribosome [17], [18]; the ribosome itself has also been shown to affect the folding process [12], [19], [20], [21]. While some proteins appear to fold by the same pathway on and off the ribosome [18], [22], other studies suggest that the folding pathways may differ [17], [23], [24], [25]. Recent studies also suggest that the process of cotranslational folding can provide a further level of protein regulation [26], [27], [28], [29].

The vectorial emergence of the peptide chain from the ribosome tunnel prompts the question of how circular permutation of a protein affects cotranslational folding. Circular permutants (CPs) of isolated domains have been extensively studied to investigate the relationship between protein topology, chain connectivity, and folding pathways [30], [31], [32], [33], [34], [35]: the permuted protein is covalently linked at the N and C termini, and new termini are generated elsewhere in the sequence, usually in a loop region [36]. This allows retention of the same amino acid composition and chain length as wild-type, but alters the connectivity of secondary structure elements; this in turn may lead to alterations in protein stability [37], [38], [39], enzyme activity [40], [41], and folding pathway [42], [43], [44].

**Fig. 1:**
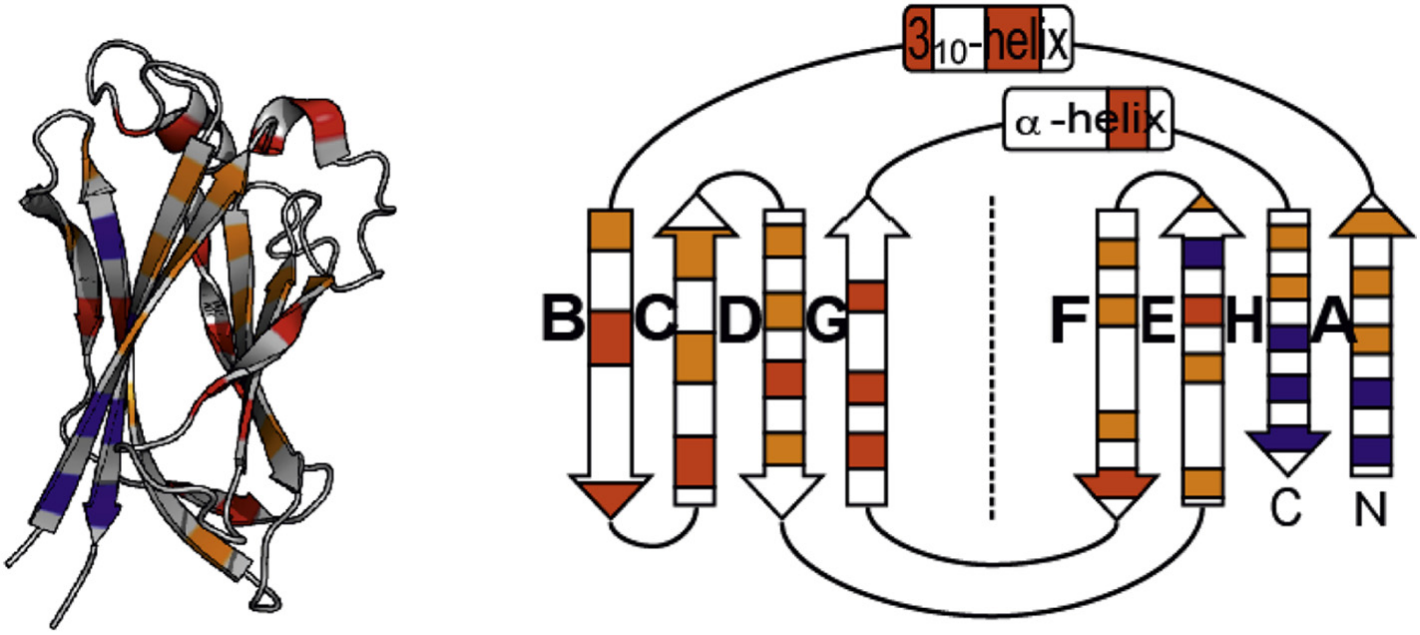
MATH wild-type structure and Φ-value analysis. (left) MATH domain with Φ-values displayed. (right) Schematic view of MATH showing the topology of the protein with Φ-values displayed. High Φ-values in blue (0.6–1), medium Φ-values in orange (0.3–0.59), and low Φ-values in red (0–0.29).

In order to study the effect of circular permutation on folding, both on and off the ribosome, we employed a MATH domain, a member of the TRAF domain-like superfamily, a β-sandwich domain containing a circularly permuted immunoglobulin (Ig)-fold topology with an extra strand [45] (Fig. 1). The N and C termini are adjacent to one another and the terminal H-strand is central to the structure. Using Φ-value analysis, we determined the folding pathway for MATH wild-type and two CPs. We also use an *in vitro* arrest-peptide-based force-measurement assay (AP assay) [46], [47], [48], to determine when MATH can begin to fold, in a stalled ribosome system, and how this is affected by changes in chain connectivity upon circular permutation.

**Fig. 2:**
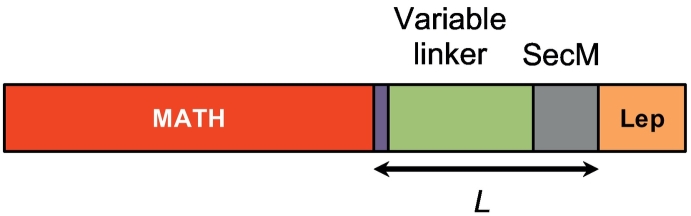
Construct used in the arrest peptide assay. MATH domain (red), SGSG linker (purple), variable linker (green), SecM stall site (gray), terminal Lep domain (orange). (Full amino acid sequences for MATH wild-type, CP-CD, and CP-DE are shown in Supplementary Information).

Translational arrest peptides [49], [50] such as SecM have been employed previously to study protein insertion into membranes and also to study cotranslational protein folding on the ribosome [14], [17], [46], [47], [48], [51]. In such assays, an amino acid linker of increasing length (*L*) is placed between the protein and the arrest site (Fig. 2); when translation is stalled by the arrest peptide, the stall can be released by the application of a moderate force, such as that generated by a protein as it folds in close proximity to the ribosome [51], [52]. The probability of release is proportional to the force applied: to release the stall, the protein must be able to fold at that force and have a sufficiently long lifetime to release the stall [51]. The fraction of full-length protein (*f*_FL_) (Fig. S1) released in a given time interval, at each value of *L*, gives insights into when folding occurs during translation.

## Results

### WT folding and analysis of the kinetic data

Kinetic data for MATH wild-type and variants show rollover in both unfolding and refolding (Fig. S2). Rollover in the refolding arm is protein concentration independent, suggesting that MATH folds *via* an early, off-pathway intermediate (see discussion in Supplementary Information). Moreover, rollover observed in the unfolding arm is consistent with a high-energy unfolding intermediate [53] (Fig. S3). Analysis of kinetic data is described in Supplementary Information. Since the refolding kinetics are complicated by the presence of the roll-over, all Φ-values were determined (Eq. (1)) using unfolding rate constants of wild-type and mutant (*k*_u_^wt^ and *k*_u_^mut^) at 3 M guanidinium chloride (GdmCl), where we had accurate data for wild-type and all mutants:

(1) $$\Phi =1-\left(\mathrm{∆∆}G_{‡-N}/\mathrm{∆∆}G_{D-N}\right)$$

where ∆∆* G*_‡-N_ = −* RT*ln(*k*_u_^wt^/*k*_u_^mut^). The change in free energy on unfolding, ∆∆* G*_D-N_, was determined using equilibrium denaturation curves.

### Terminal strands A and H are most structured region in MATH transition state

A Φ-value analysis was performed to elucidate the folding pathway of wild-type MATH: 39 mutations at 37 separate locations were made across the protein (Table S1). Only mutations that were sufficiently destabilizing (ΔΔ*G*_D-N_ > 0.75 kcal mol^−1^) [54] were considered for Φ-value analysis.

Except for the cross-sheet helical regions and strands B and G, Φ-values are all > 0, suggesting that most regions of MATH are at least partially structured in the transition state for folding (Fig. 1 and Table S1). We find the highest Φ-values are in the N and C terminal strands, which includes the peripheral A-strand (N-terminus) and structurally central H-strand (C-terminus). Overall, residues in the sheet containing strands F, E, H, and A have higher Φ-values than those in the sheet containing strands B, C, D, and G (Fig. 1 and Table S1). The most structured region of the transition state for folding in wild-type MATH is located in the area around the N- and C-terminal strands (strands A and H, respectively).

### Circular permutation increases both folding and unfolding rates

**Fig. 3:**
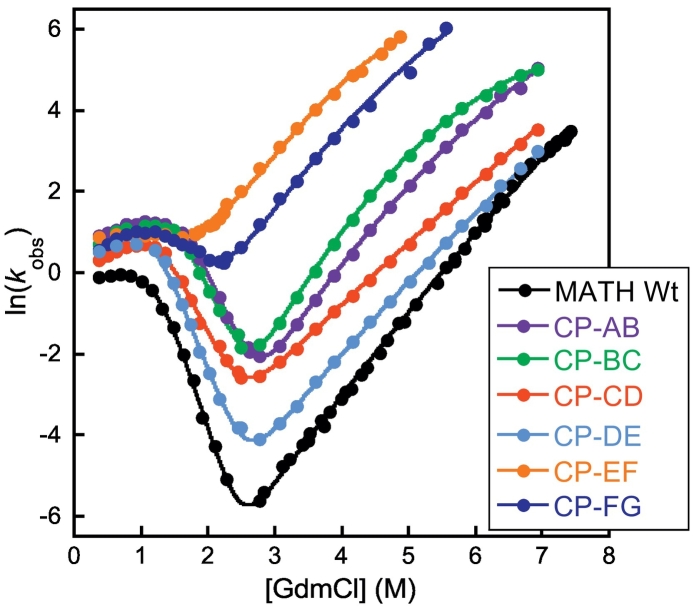
Kinetic data for MATH wild-type and CPs. All MATH permutants fold and unfold more rapidly than wild-type.

To determine the effects of circular permutation upon the stability and folding and unfolding rates of MATH, a series of CPs were made. The N and C termini were joined (with no additional residues added) and new N and C termini were located in each of the seven loops and turns (see Materials and Methods and Fig. S4 for details). All CPs were designed to disrupt the native connectivity of the wild-type domain while minimizing the potential impact on native structure and topology; we use the nomenclature CP-XX, where XX denotes the position of the new N and C termini (thus, CP-AB has new termini in the A–B loop). We characterized the stability and kinetics of six of these CPs (Table S2). The exception is CP-GH, which could not be produced solubly. All CPs are stable and, like wild-type, fold in a reversible two-state manner at equilibrium; all CPs showed rollover in the refolding arm, as observed in wild-type MATH (Fig. 3). Most significantly, our chevron plots reveal that both the folding and unfolding rate constants of MATH are increased in all permutants.

### CP-CD and CP-DE display a similar pattern of Φ-values to wild-type

**Fig. 4:**
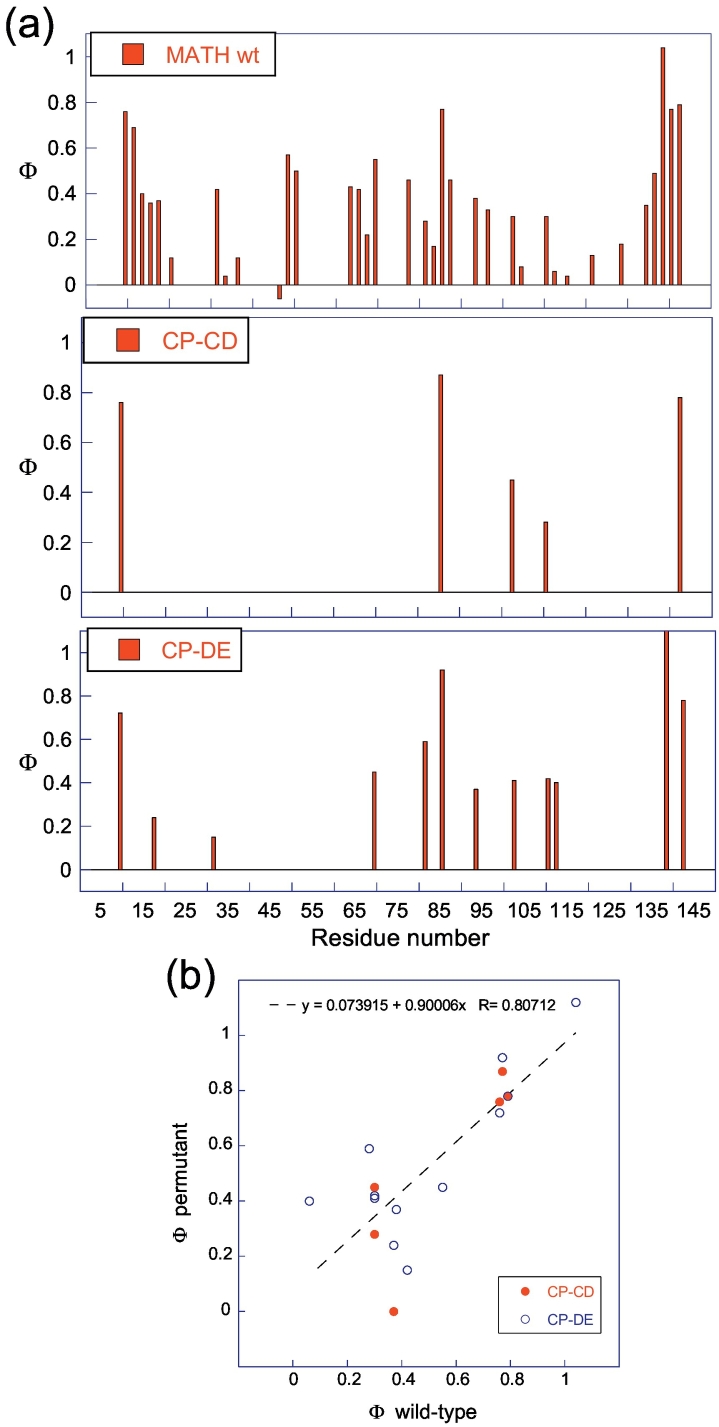
Comparison of Φ-values for MATH wild-type, CP-CD, and CP-DE. (a) Histograms of Φ-values for MATH wild-type (top), CP-CD (center), and CP-DE (bottom). (b) A plot of Φ-values for MATH wild-type *versus* Φ-values for CPs showing a linear fit of the data, which gives an *R*-value of 0.81.

In order to determine how changes in connectivity affect the folding pathway of MATH, partial Φ-value analyses were performed on two of the permutants, CP-CD and CP-DE (Fig. 4, Tables S3 and S4). These permutants were selected as new termini are generated in the center of the BCDG sheet in CP-CD, whereas new termini are generated between the two β-sheets in CP-DE (Fig. S4); also, both permutants are stable and easy to produce and have linear unfolding arms (Fig. 3). A number of conservative mutations were made throughout the core of the proteins to probe tertiary structure formation and, where possible, matched to mutations made previously in wild-type. As for wild-type MATH, Φ-values were determined using unfolding rate constants at 3 M GdmCl and equilibrium values of ∆∆* G*. For CP-CD, six variant proteins could be produced, which were sufficiently destabilized for Φ-value analysis; a further nine variants were found to be insoluble and could not be isolated, seven of which contained a mutation in the BCDG sheet. We observed that mutations in CP-CD are less destabilizing on average than equivalent mutations in wild-type, except V10A (A-strand) and V143A (H-strand), which are destabilized by a similar amount (Table S3). A comparison with wild-type shows that high Φ-values in the A- and H-strands are maintained in CP-CD (Fig. 4). For CP-DE, 12 variant proteins were sufficiently destabilized for Φ-value analysis; only two mutations, located in strands D and H, resulted in insoluble protein, which could not be isolated. Three variants with mutations located in the D, F, and G strands (L70A, Y103A, and W111L, respectively) are destabilized to a greater extent than in wild-type; however, mutations in the A, B, and H strands have a similar effect on stability (Table S4). CP-DE also displays a pattern of Φ-values similar to that of wild-type, with high values in the A- and H-strands (Fig. 4). The most structured region of the transition state in both CPs is maintained in the FEHA sheet and particularly around the terminal strands A and H, as in wild-type MATH.

### MATH folds close to the ribosome

**Fig. 5:**
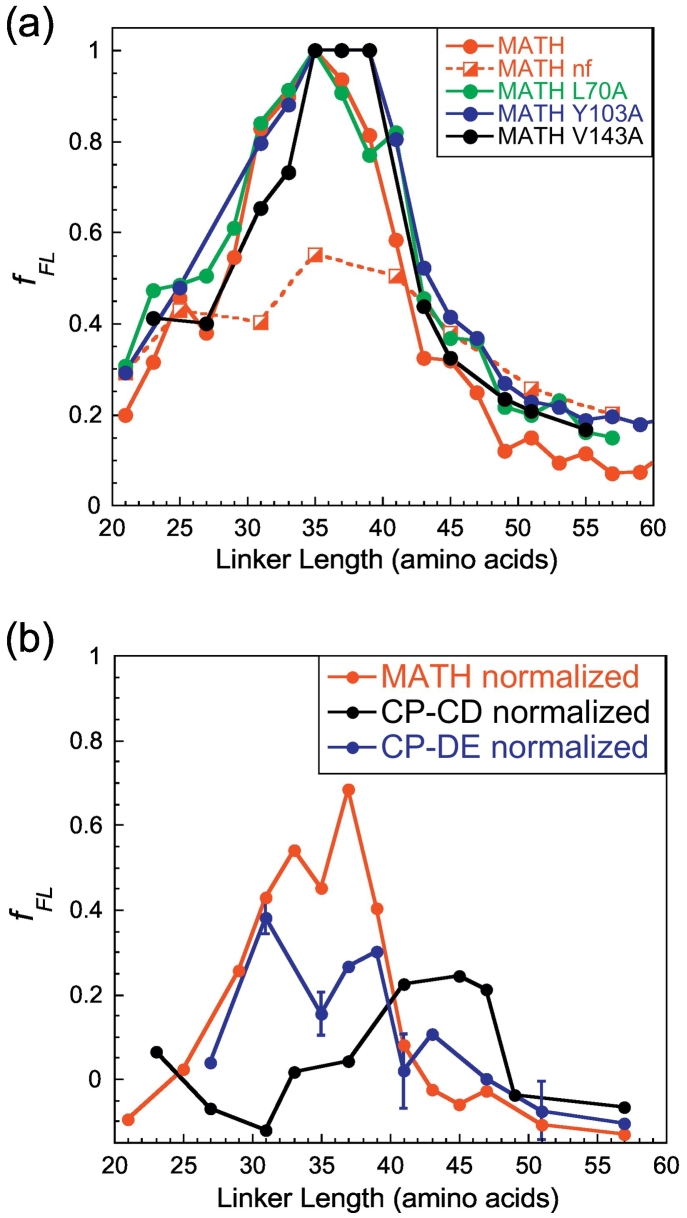
Following folding of MATH on the ribosome using the arrest peptide assay. (a) Force profiles of wild-type MATH with non-folding controls MATH (W16G/W47G/F84E) (red) and three variants MATH L70A (green), Y103A (blue), and V143A (black). The small differences between the four force profiles reflect the reproducibility expected from such assays as reported previously [17]. (b) Force profiles of CP-CD and CP-DE compared with wild-type MATH normalized against non-folding controls. Data points shown at 14 linker lengths for wild-type MATH (red) are normalized against the nf control MATH (W16G/W47G/F84E); data shown at 10 linker lengths for CP-CD (black) are normalized against the nf control CP-CD (W16G/W47G/F84E); data shown at 10 linker lengths for CP-DE (blue) are normalized against the nf control CP-DE (W16G/W47G/F84E). Error bars are shown at linker lengths for CP-DE where experiments (sample plus nf control) were performed in triplicate.

Can cotranslational folding begin before the entire MATH domain is fully emerged from the ribosome? To investigate this, we used an *in vitro* AP assay, as described in Refs. [14], [17] to determine when wild-type MATH can commence folding on the ribosome (Fig. 5a). In the study we report here, we use this assay to determine when the MATH domain can fold productively by assessing from the force profile at what linker length a force is generated sufficient to release the stall. In addition to obtaining force-profile results for wild-type MATH, we also performed arrest peptide experiments on a non-folding (nf) variant of MATH (W16G/W47G/F84E) at 14 different linker lengths; this is to ensure that fraction full-length values (*f*_FL_) obtained are due to a folding event and not due to non-specific interactions with the ribosome tunnel. Previous studies have estimated that the ribosome tunnel can sequester in the range of 30–40 amino acids [12], [55], [56]. Our results show that MATH can release the stall at linker length (*L*) = 31 with a force peak extending to (*L*) = 39 (Fig. 5a), suggesting that MATH can fold cotranslationally when a proportion of C-terminal (H-strand) residues are still located in the ribosome tunnel. Three variants of wild-type MATH were also employed in the AP assay, each destabilized by 2–3 kcal mol^−1^ relative to wild-type (Table S1): L70A (D-strand) and Y103A (F-strand) are centrally located, and V143A is located in the C-terminal H-strand. All three variants exhibit a force-measurement profile similar to wild-type (Fig. 5a) showing that a reduction in intrinsic stability of this magnitude does not influence the cotranslational folding of MATH close to the ribosome. (We define “intrinsic” as relating to the properties of the native domain in isolation).

### Onset of folding does not correlate with location of strands A and H in the ribosome tunnel

As residues with the highest Φ-values are found in the terminal strands A and H in wild-type MATH, CP-CD, and CP-DE, we ask: does the location of these strands in the ribosome tunnel influence the onset of cotranslational folding? To investigate this, we employed CP-CD and CP-DE in the AP assay. Very importantly, each of these CPs, although precisely the same length as wild-type MATH (see Fig. S5a), with precisely the same leader, variable linker and SecM sequence, will have different residues interacting with the ribosome tunnel at any given linker length; to control for this, we made non-folding mutants of each CP as well; thus, in the results, the data for each CP is shown normalized against *its own* non-folding variant (Fig. 5b).

CP-CD has new termini located in the middle of the BCDG sheet, central to the structure, and is destabilized by approximately 2 kcal mol^−1^ compared with the isolated wild-type domain (Table S2); the H- and A-strands will emerge from the ribosome after strands D, E, F, and G, but before strands B and C (Fig. S5b). Our data show that the force profile of CP-CD results in a later force peak compared with wild-type (Fig. 5b). We infer that for this permutant, folding does not occur until most or all of the domain has emerged from the ribosome tunnel.

The circular permutant CP-DE has new termini located between the two β-sheets and has a similar stability to wild-type (Table S2). In this permutant, strands H and A are located in the center of the protein (Fig. S5). Force-profile data for CP-DE show that folding occurs at a similar linker length to wild-type, *L* = 31, and also like wild-type, the force peak extends to *L* = 39 (Fig. 5b).

Taken together, our data show that the earlier emergence of the H–A-strand pair during translation does not result in earlier onset of productive cotranslational folding in the CPs.

## Discussion

We use a MATH domain and two CPs to investigate how alterations in chain connectivity affect the folding pathway and the onset of folding on the ribosome. The generation of CPs allows the investigation of the folding of MATH variants with identical amino acid sequences, but different chain connectivity and order of secondary structural elements.

Our Φ-value analysis of MATH wild-type shows that folding proceeds by a nucleation-condensation mechanism, in which secondary and tertiary structure form concomitantly. Previous experimental and simulation studies suggest that a folding nucleus can be subdivided into “obligate” and “critical” nuclei [57], [58]: Residues that constitute the obligate nucleus interact early to establish the correct topology of the protein; these in turn are supported by a shell of additional interactions, which constitute the critical nucleus, turning the free energy profile downhill. Φ-values report upon the extent of structure formation in the transition state, but not the order in which that structure is formed in that nucleus [59]. Our data are consistent with the A-strand being almost fully structured in the transition state, and thus, we infer, fully packed onto the terminal H strand (Fig. 1). However, it seems unlikely that interaction of the N and C terminal strands alone would be sufficient to set up the complex topology of the MATH domain fold. Instead we propose that residues with relatively high Φ-values in strands C, D, E, and F interact to form an obligate folding nucleus, setting up the complex topology of the domain and establishing the correct register of the strands and the packing of the two sheets. This nucleus would then be further stabilized by the packing of the peripheral strands A and H (which in this case constitute the critical nucleus). In this model, formation of interactions between residues in strands A and H is energetically critical—the N and C terminal regions of the protein must come together and pack to provide sufficient energy to cross the transition state barrier, resulting in high Φ-values in this region of the protein.

There have been a number of studies of Greek-key proteins that have given support to an obligate-critical nucleus model such as that we propose here: The Ig domain titin I27 (I27) and the evolutionarily unrelated fnIII domains, TNfn3, FNfn10, and CAfn2 share a structurally equivalent obligate nucleus, comprising key residues located in the four central strands, which establishes the topology of these complex Greek-key domains [60], [61], [62], [63], [64]. Similar results were seen for the all-beta Greek key protein S6, and for an all-alpha Greek key death domain [35], [43].

Upon circular permutation of MATH, the terminal A- and H-strands are covalently linked and become adjacent in the primary sequence. We thus predicted that connection of the terminal strands and generation of new N and C termini in loop regions would increase the folding rate of the CPs due to a reduction in the entropic cost of bringing the terminal strands together during folding [65]. Indeed we find that all six CPs exhibited an increased rate of both folding and unfolding, indicating that the energetic cost of forming the transition state is lower in the permutants. In most previous studies, circular permutation has been shown to alter the folding pathway of a protein [42], [43], [44] such as in CPs of S6 [30] and the SH3 domain of α-spectrin [66], which have a shifted folding nucleus upon changes in connectivity. However, the pattern of Φ-values seen in wild-type MATH is essentially maintained in CP-CD and CP-DE (Fig. 4): our data are consistent with the hypothesis that these permutants follow a similar folding pathway to wild-type.

The question of when cotranslational folding can begin on the ribosome is of particular interest in the case of MATH: Due to the vectorial synthesis of the peptide chain, the A-strand will emerge first in wild-type and be outside the ribosome, while the H-strand is still sequestered in the tunnel. Our Φ-value analysis shows that interactions between strands H and A are energetically critical for folding. As shown in Fig. 1, the A-strand cannot pack onto the protein until the H-strand is formed, so we expected that the entire domain of MATH would need to be fully emerged before folding can occur. However, wild-type MATH and three destabilized variants (Fig. 5a) can fold cotranslationally, while a proportion of C-terminal residues are still sequestered in the ribosome.

We also predicted that CP-CD and CP-DE may fold earlier on the ribosome compared with wild-type, as the H-strand will emerge earlier from the tunnel (Fig. S5). The onset of the folding is revealed in the arrest peptide assay, and we see that neither permutant folds earlier than wild-type in our cotranslational folding assay (Fig. 5b). For CP-DE, folding occurs at the same linker length as wild-type, whereas CP-CD can fold only when fully emerged from the ribosome. We can rule out loss of intrinsic stability as being key to the failure of CP-CD to fold close to the ribosome, as it has a similar stability to the wild-type variants Y103A and V143A.

It appears that in the case of MATH, cotranslational folding is not influenced by formation of interactions between strands A and H. Instead we propose that formation of both β-sheets, critical to formation of the obligate nucleus, is required for formation of sufficiently stable structures to be able to fold close to the ribosome. In CP-DE, connectivity within each sheet is maintained as the new termini are created within a cross-sheet loop. However, in CP-CD, the new termini created in strands C and D disrupt the connectivity of the BCDG sheet. We infer that the BCDG sheet in CP-CD is now too destabilized to contribute to formation of the obligate nucleus, until the protein is fully emerged from the ribosome.

We have shown that chain connectivity can influence the onset of productive cotranslational folding of a MATH domain on the ribosome, but not in a way we initially predicted. In the CPs, the earlier emergence of residues which are the most highly structured in the rate-limiting transition state, in the isolated domains, does not result in folding occurring earlier on the ribosome. Taking all our data together, we propose that stable formation of an obligate nucleus (likely similar to that seen in our folding studies of isolated domains and involving strands C, D, E, and F) is required for the folding of MATH to be productive on the ribosome. Small all β-domains often form part of larger multi-domain proteins; we speculate that MATH is unable to fold on the ribosome until both β-sheets are formed and able to pack, to prevent the accumulation of partly folded structures which could lead to misfolding or aggregation.

## Materials and Methods

### DNA manipulation for expression of isolated domains

MATH wild-type (PBD:2CR2 residues 4–153) and circularly permuted constructs were generated as synthetic genes (Life Technologies) and cloned into pRSET A plasmid (Invitrogen) (previously modified to remove the sequence including the entire T7 gene 10 leader and EK recognition site up to, but not including, the BamH I site and replaced with a sequence encoding residues GLVPRGS).

CPs are given the nomenclature CP-XX, where XX denotes the position of the new termini. In each case, the residues DSGK (shown in the wild-type sequence below in bold) were used to form a linker connecting the original N and C-termini, and new termini are generated in a loop region. For the isolated constructs of all CPs, a glycine followed by residues SVN (shown underlined in the wild-type sequence below) were added to the newly generated C-terminus, as this was found to improve solubility. For CP-DE, an additional C73A mutation was made at the new C-terminus to avoid a free Cys residue. The amino acid sequence of MATH wild-type is as follows:

GS**SGK**VVKFSYMWTINNFSFCREEMGEVIKSSTFSSGANDKLKWCLRVNPKGLDEESKDYLSLYLLLVSCPKSEVRAKFKFSILNAKGEETKAMESQRAYRFVQGKDWGFKKFIRRDFLLDEANGLLPDDKLTLFCEVSVVQ**D**SVNISGQ.

### Protein expression and purification

Protein was expressed using *Escherichia coli* C41 in 2 × YT containing 100 μg mL^−1^ IPTG at 37 °C with shaking. Cells were grown overnight at 22 °C and then harvested by centrifugation at 6000 rpm (Sorvall RC-5C). The cell pellets were resuspended in 1 × PBS (pH 7.4) and 2 mM β-mercaptoethanol and lysed by sonication. Cell lysate was cleared by centrifugation at 18,000 rpm (Sorvall RC-5C), and the soluble protein supernatant was bound to Ni^2+^ agarose resin (Agarose Bead Technologies). Resin was then washed four times in 1 × PBS (pH 7.4) and then resuspended in 50 mM Tris (pH 8.2), and protein was cleaved from the resin overnight at 25 °C using thrombin (Sigma). Protein was further purified by gel filtration chromatography using a HiLoad Superdex G75 column (GE Healthcare) equilibrated in 1 × PBS (pH 7.4).

### Equilibrium denaturation and kinetics studies

Stability of proteins was determined by GdmCl denaturation in 1 × PBS (pH 7.4) containing up to 10 mM DTT at 25 °C using the intrinsic fluorescence of tryptophan after excitation at 280 nm. Emission spectra between 300 and 400 nm were recorded using a PerkinElmer LS-55 or a Cary Eclipse fluorimeter. Final protein concentration was 1 μM. The kinetics of protein folding and unfolding was measured in GdmCl in 1 × PBS (pH 7.4) containing up to 10 mM DTT at 25 °C using intrinsic tryptophan fluorescence. Proteins were excited at 280 nm using an Applied Photophysics SX.18 or SX.20 stopped-flow spectrophotometer, and the fluorescence emission was collected above 320 nm. The final protein concentration was 1 μM, and six traces were averaged for each concentration of denaturant. Final protein concentrations of 0.1, 1.0, and 2 μM for MATH wild-type and 0.1, 1.0, and 6.5 μM for CP-DE were used to demonstrate protein concentration-independent rollover below 2 M GdmCl. Data were fitted to a single-exponential model using KaleidaGraph (Synergy Software). For analysis of kinetic data, see Supplementary Information.

### DNA manipulations for AP assays

A plasmid was generated containing the *E. coli* SecM arrest peptide (FSTPVWISQAQGIRAGP) [67], [68] and a terminal Lep segment (23 amino acids) derived from leader peptidase [46] in a modified version of pRSET A (Invitrogen). Increasing linker lengths (from *L* = 21 to *L* = 61 in increments of 2 amino acids) were generated in the vector using site-directed mutagenesis. DNA templates for *in vitro* transcription–translation were prepared using the In-Fusion cloning system (Takara Bio). Genes of interest were amplified by PCR with overhanging homology to the vector and subsequently cloned into pRSET A containing the appropriate linker length, using the In-Fusion system. All constructs were verified by DNA sequencing. For MATH wild-type site-directed mutagenesis was performed to generate non-folding triple mutant controls (W16G/W47G/F84E) at 14 linker lengths (*L* = 21, 25, 29–47, 51, and 57). For MATH CPs, site-directed mutagenesis was performed to generate non-folding triple-mutant controls (W16G/W47G/F84E) at 10 linker lengths for CP-CD (*L* = 23, 27, 31, 33, 37, 41, 45, 47, 49, and 57) and for CP-DE (*L* = 27, 31, 35–43, 47, 51, and 57). Site-directed mutagenesis was also performed on wild-type MATH to generate constructs with the non-functional FSTPVWISQAQGIRAGA arrest peptide (P to A mutation underlined) as full-length controls; also arrested controls were generated by insertion of a stop codon directly after the arrest peptide. Protein sequences of constructs used in the AP assays for MATH wild-type, CP-DE, and CP-CD (*L* = 61) are shown in Supplementary Information (Fig. S5a).

### *In vitro* transcription and translation

Transcription and translation were performed using the PUREfrex cell-free translation system according to the manufacturer's instructions (GeneFrontier Corporation) [69], [70]. ^35^S-Methionine (PerkinElmer) was added to a master mix containing all components of the PUREfrex system and subsequently added to pre-prepared samples of template DNA (250 μg). Synthesis of labeled polypeptides was performed for 15 min at 37 °C with shaking at 500 rpm. Reactions were quenched using an equal volume of ice-cold 10% (v/v) trichloroacetic acid on ice for 30 min. Labeled polypeptides were pelleted by centrifugation at 14,440*g* for 5 min at 4 °C. The supernatant was discarded, and the pellet was resuspended in 1 × LDS buffer (Life Technologies) with shaking at 14,000 rpm, 15 min at 37 °C. RNA was digested with RNase A (ThermoFisher Scientific) with shaking at 700 rpm for 15 min at 37 °C. Samples were resolved by SDS-PAGE and fixed for 1 h using Instant Blue protein stain (Expedeon), and gels were dried for 2 h. Images were transferred to a phosphor screen for 2 days before imaging. Bands were quantified using ImageJ (http://rsb.ingo.nih.gov/ij/) and fit to a Gaussian distribution (Fig. S1c) using KaleidaGraph (Synergy Software). To obtain normalized fraction full-length values (*f*_FL_), the *f*_FL_ value for the nf control was subtracted from the *f*_FL_ value for the equivalent sample. For MATH wild-type, normalized data points in the force-measurement profiles are single experiments; for CP-CD, normalized data points are from a single experiment except for *L* = 41 and 45, which are an average of two experiments. For CP-DE, normalized data points are from a single experiment except *L* = 27, which is an average of two experiments, and *L* = 31, 35, 41, and 51, which are an average of at least three experiments. For MATH wild-type variants L70A, Y103A, and V143A, data points are not normalized and are from a single experiment. The reproducibility of data obtained from arrest peptide force measurement assays has been discussed previously [17].

## Appendix A Supplementary data

Supplementary material Image 1
