# Supplementary material for: Investigating the Effect of Chain Connectivity on the Folding of a Beta-Sheet Protein On and Off the Ribosome
Source: J Mol Biol. 2018 Dec 7;430(24):5207–16. doi: 10.1016/j.jmb.2018.10.011 (PMC6288478; doi:10.1016/j.jmb.2018.10.011)
Supplement: Supplementary file 1 — Supplementary material [file mmc1.pdf]

Supplementary information

Analysis of chevron plots for MATH wild-type and variants, and circular permutants

Kinetic data for wild-type MATH and variants show rollover in both unfolding and refolding (Fig. S2); rollover in the refolding arm is also observed in all six circular permutants (Fig. 3). We determined for wild-type MATH, and also for CP-DE, that rollover in the refolding arm is independent of protein concentration (see Materials and Methods); we therefore make the assumption that rollover is concentration independent for all MATH proteins and thus not due to protein aggregation, but due to the presence of a folding intermediate [1]; since the *m*-value for this intermediate is negative, we also make the assumption that the intermediate is off-pathway [2,3]. For the Φ-value analysis of wild-type MATH, unfolding rate constants obtained at 3.0 M GdmCl were used to calculate Φ-values, in order to avoid a long extrapolation to 0 M denaturant. Rollover observed in the unfolding arm is consistent with a high energy intermediate (Fig. S3) [4,5]. The procedure used in fitting chevrons to a sequential transition state model with a high-energy intermediate has been described previously [4]. Observed rate constants for MATH were calculated from a global fit of wild-type and variants with shared *m*-values, using a modified version of the sequential transition state model, in which a low energy intermediate is included (shown as off-pathway in Fig. S3). Global fitting performed using Prism (GraphPad Software).

The observed rate constant is:

$$\ln(k_{\text{obs}}) = \ln\left[0.5\left(-\mathrm{A}_1 - \sqrt{\mathrm{A}_1{}^2 - 4.\mathrm{A}_2}\right)\right]$$

where:

$$\mathrm{A}_1 = -\left\{k_1\mathrm{e}^{(-m_1[\mathrm{Den}])} + k_{-1}\mathrm{e}^{(m_{-1}[\mathrm{Den}])} + k_2\mathrm{e}^{(-m_2[\mathrm{Den}])} + k_{-2}\mathrm{e}^{(m_{-2}[\mathrm{Den}])}\right\}$$

$$A_2 = \{k_1 e^{(-m_1[\text{Den}])}.[k_2 e^{(-m_2[\text{Den}])} + k_{-2} e^{(-m_{-2}[\text{Den}])}] + k_{-1} e^{(m_{-1}[\text{Den}])}.k_{-2} e^{(m_{-2}[\text{Den}])}\}$$

To compare kinetic chevron data of wild-type MATH with those of the circular permutants, chevron data for the CPs were fitted individually to the modified sequential transition state model, allowing *m*-values to vary.

NB The analysis of kinetic data presented in this work does not depend upon the assumption that the refolding intermediate is off-pathway.

Φ-value analysis

Since the refolding kinetics are complicated by the presence of the roll-over, all Φ-values were determined using unfolding rate constants of wild type and mutant (k_u^{wt} & k_u^{mut}) at 3 M guanidinium chloride (GdmCl), where we had accurate data for wild-type and all mutants:

$$\Phi = 1 - (\Delta\Delta G_{\ddagger\text{-N}} / \Delta\Delta G_{\text{D-N}})$$

where $\Delta\Delta G_{\ddagger\text{-N}} = -RT\ln(k_u^{wt} / k_u^{mut})$.

The change in free energy on unfolding, $\Delta\Delta G_{\text{D-N}}$, was determined using equilibrium denaturation curves.

For the partial Φ-value analyses of CP-CD and CP-DE, unfolding rate constants obtained at 3.0 M GdmCl were again used to calculate Φ-values. Only unfolding data, not full chevron plots were collected for these CP variants. For comparison of CP-CD and CP-DE with their respective variants, unfolding data were fitted globally using a single shared *m*-value, with a linear dependence of $\ln(k_{obs})$ on GdmCl concentration. For three variants of CP-DE (F82L, I86A and T94G) unfolding data could not be fitted globally due to curvature in the unfolding arm; for these variants individual Hammond fits were used [6].

References

[1] M. Silow, M. Oliveberg, Transient aggregates in protein folding are easily mistaken for folding intermediates, Proc. Natl. Acad. Sci. U.S.A. 94 (1997) 6084-6086.

[2] A. Matouschek, J.T. Kellis, L. Serrano, M. Bycroft, A.R. Fersht, Transient folding intermediates characterized by protein engineering, Nature 346 (1990) 440-445.

[3] R.L. Baldwin, On-pathway versus off-pathway folding intermediates, Folding and Design 1 (1996) R1-R8.

[4] A. Bachmann, T. Kiefhaber, Apparent two-state tendamistat folding is a sequential process along a defined route, J. Mol. Biol. 306 (2001) 375-386.

[5] W.F. Walkenhorst, S.M. Green, H. Roder, Kinetic evidence for folding and unfolding intermediates in staphylococcal nuclease, Biochemistry, 36 (1997) 5795-5805.

[6] I.E. Sánchez, T. Kiefhaber, Hammond behavior versus ground state effects in protein folding: evidence for narrow free energy barriers and residual structure in unfolded states, J. Mol. Biol. 327 (2003) 867–884.

Supplementary Tables

Table S1: Thermodynamic and kinetic parameters for MATH wild-type

**Mutation**	**Location**	$\Delta G_{D\text{-}N}$ (kcal mol^{-1}) [a]	$k_u^{3.0\ M}$ (s^{-1}) [b]	$\Phi_U^{3.0\ M}$ [c]
WT	-	11.51 ± 0.06	0.0061	-
V10A	Strand A	10.61 ± 0.06	0.0089	0.76
F12A	Strand A	10.19 ± 0.06	0.0122	0.69
Y14A	Strand A	9.50 ± 0.05	0.0467	0.40
W16A	Strand A	8.15 ± 0.06	0.227	0.36
I18V	Strand A	9.87 ± 0.06	0.0351	0.37
F21A	3_{10}-helix	8.94 ± 0.05	0.277	0.12
I32V	Strand B	10.24 ± 0.05	0.0213	0.42
S34A	Strand B	8.33 ± 0.05	1.07	0.04
F37L	Strand B	8.70 ± 0.05	0.406	0.12
W47L	Strand C	8.15 ± 0.05	2.54	-0.06
L49A	Strand C	8.84 ± 0.04	0.0431	0.57
V51G	Strand C	8.94 ± 0.04	0.0528	0.50
L64A	Strand D	8.70 ± 0.04	0.0911	0.43
L66G	Strand D	7.31 ± 0.04	0.363	0.42
L68A	Strand D	7.73 ± 0.05	0.911	0.22
L70A	Strand D	8.52 ± 0.04	0.0603	0.55
V78A	Strand E	9.64 ± 0.05	0.0341	0.46
F82L	Strand E	8.75 ± 0.04	0.179	0.28
F84L	Strand E	9.87 ± 0.05	0.0623	0.17
I86A	Strand E	9.26 ± 0.05	0.0149	0.77

N88A	Strand E	9.31 ± 0.05	0.0456	0.46
T94G	Strand F	10.47 ± 0.05	0.0181	0.38
M97A	Strand F	10.33 ± 0.05	0.0231	0.33
Y103A	Strand F	9.45 ± 0.05	0.0714	0.30
F105L	Strand F	8.47 ± 0.07	0.687	0.08
W111L	Strand G	10.05 ± 0.05	0.0341	0.30
F113L	Strand G	8.38 ± 0.04	0.891	0.06
F116L	Strand G	8.33 ± 0.05	1.05	0.04
L122A	α-helix & loop	8.10 ± 0.06	0.918	0.13
L129A	α-helix & loop	9.45 ± 0.05	0.107	0.18
L135A	Strand H	7.31 ± 0.06	0.627	0.35
L137A	Strand H	7.49 ± 0.11	0.198	0.49
C139A	Strand H	10.52 ± 0.05	0.0058	1.04
V141A	Strand H	9.08 ± 0.05	0.0157	0.77
V143A	Strand H	9.59 ± 0.05	0.0122	0.79

[a] $\Delta G_{D\text{-}N}$ was calculated using a mean $m_{D\text{-}N}$ value of 4.66 ± 0.02 kcal mol^{-1} M^{-1} from a global fit of wild-type and all mutants.

[b] Unfolding rate constants at 3.0 M GdmCl were calculated from a global kinetic fit of wild-type and mutants. The error in $k_u^{3.0M}$ is ±10 %.

[c] Φ-values calculated at 3.0 M GdmCl report on structure formation in the major rate-limiting transition state. The error in $\Phi_U^{3.0M}$ is ±0.1.

Table S2: Thermodynamic and kinetic parameters for MATH circular permutants

Construct	$\Delta G_{D\text{-}N}$ (kcal mol^{-1}) [a]	$k_u^{0\,M}$ (s^{-1}) [b]
WT	11.51 ± 0.06[c]	1.04 × 10^{-5}
CP-AB	11.39 ± 0.05	2.20 × 10^{-4}
CP-BC	11.75 ± 0.28	1.12 × 10^{-4}
CP-CD	9.46 ± 0.44	1.34 × 10^{-3}
CP-DE	11.41 ± 0.12	1.23 × 10^{-4}
CP-EF	8.06 ± 0.32	3.49 × 10^{-2}
CP-FG	7.12 ± 0.12	1.72 × 10^{-2}

[a] $\Delta G_{D\text{-}N}$ values for the circular permutants were calculated from individual fits using the following $m_{D\text{-}N}$ values: CP-AB, 4.15 ± 0.10 kcal mol^{-1} M^{-1}; CP-BC, 4.32 ± 0.10 kcal mol^{-1} M^{-1}; CP-CD, 3.69 ± 0.17 kcal mol^{-1} M^{-1}; CP-DE, 4.24 ± 0.10 kcal mol^{-1} M^{-1}; CP-EF, 3.28 ± 0.13 kcal mol^{-1} M^{-1}; CP-FG, 3.06 ± 0.10 kcal mol^{-1} M^{-1}.

[b] Unfolding rate constants at 0 M GdmCl were calculated from an individual fit of each chevron.

[c] $\Delta G_{D\text{-}N}$ for wild-type is calculated using a mean $m_{D\text{-}N}$ value of 4.66 ± 0.02 kcal mol^{-1} M^{-1} from a global fit of wild-type and all mutants.

Table S3: Thermodynamic and kinetic parameters for MATH CP-CD

**Mutation**	**Location**	$\Delta G_{D\text{-}N}$ **(kcal mol^{-1})** [a]	$k_u^{3.0\,M}$ **(s^{-1})** [b]	$\Phi_U^{3.0\,M}$ [c]
CP-CD	-	9.29 ± 0.09	0.110	-
V10A	Strand A	8.34 ± 0.07	0.158	0.76
I18V	Strand A	8.45 ± 0.07	0.418	0.00
I32V	Strand B	9.03 ± 0.08	0.247	NM[d]
V51A	Strand C	8.67 ± 0.05	0.092	NM[d]
I86A	Strand E	7.96 ± 0.07	0.148	0.87
T94G	Strand F	9.02 ± 0.08	0.307	NM[d]
Y103A	Strand F	6.84 ± 0.05	1.030	0.45
W111L	Strand G	7.88 ± 0.05	0.582	0.28
C139A	Strand H	8.74 ± 0.07	0.093	NM[d]
V143A	Strand H	7.41 ± 0.06	0.219	0.78

[a] $\Delta G_{D\text{-}N}$ was calculated using a mean $m_{D\text{-}N}$ value of 3.60 ± 0.02 kcal mol^{-1} M^{-1} from a global fit of MATH CP-CD and all mutants.

[b] Unfolding rate constants at 3.0 M GdmCl were calculated from a global kinetic fit of MATH CP-CD and all mutants. The error in $k_u^{3.0M}$ is ±10 %.

[c] Φ-values calculated at 3.0 M GdmCl report on structure formation in the major rate-limiting transition state. The error in $\Phi_U^{3.0M}$ is ±0.1.

[d]NM – not measured; Φ-values were not determined where $\Delta\Delta G_{D\text{-}N}$ was less than 0.75 kcal mol^{-1}.

Table S4: Thermodynamic and kinetic parameters for MATH CP-DE

Mutation	Location	$\Delta G_{D\text{-}N}$ (kcal mol^{-1}) [a]	$k_u^{3.0\,M}$ (s^{-1}) [b]	$\Phi_U^{3.0\,M}$ [c]
CP-DE	-	11.25 ± 0.09	0.024	-
V10A	Strand A	10.13 ± 0.08	0.041	0.72
I18V	Strand A	9.85 ± 0.08	0.144	0.24
I32V	Strand B	10.48 ± 0.08	0.071	0.15
L70A	Strand D	7.18 ±0.16	1.050	0.45
F82L	Strand E	6.55 ± 0.16	0.609	0.59
I86A	Strand E	6.37 ± 0.07	0.045	0.92
T94G	Strand F	10.46 ± 0.09	0.056	0.37
Y103A	Strand F	8.51 ±0.06	0.367	0.41
W111L	Strand G	8.47 ±0.06	0.363	0.42
F113L	Strand G	6.53 ±0.14	2.770	0.40
C139A	Strand H	10.50 ± 0.09	0.021	1.12
V143A	Strand H	9.23 ± 0.08	0.051	0.78

[a] $\Delta G_{D\text{-}N}$ was calculated using a mean $m_{D\text{-}N}$ value of 4.23 ± 0.02 kcal mol^{-1} M^{-1} from a global fit of MATH CP-DE and mutants (excluding F82L and I86A which have lower $m_{D\text{-}N}$ values than CP-DE).

[b] Unfolding rate constants at 3.0 M GdmCl were calculated from a global kinetic fit of MATH CP-DE and mutants (excluding F82L, I86A, T94G (see supplementary information)). The error in $k_u^{3.0M}$ is ±10 %.

[c] Φ-values calculated at 3.0 M GdmCl report on structure formation in the major rate-limiting transition state. The error in $\Phi_U^{3.0M}$ is ±0.1.

Supplementary Figures:

(a)

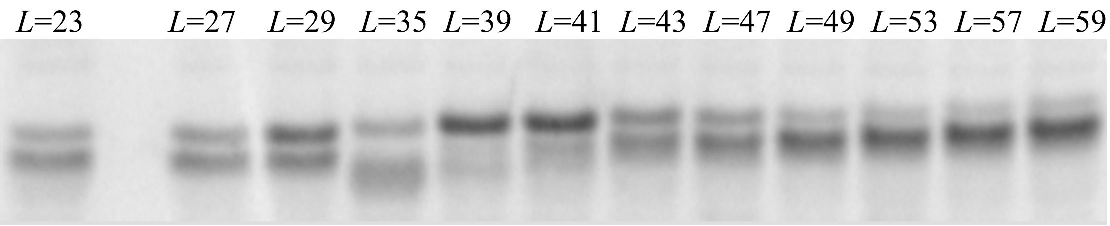

(b)

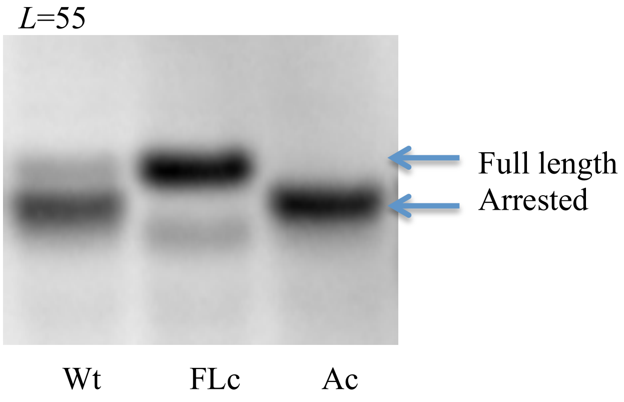

(c)

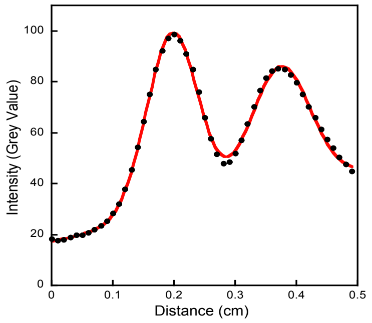

Fig. S1. *In vitro* translation using the PUREfrex cell-free translation system (a) MATH wild-type samples resolved by SDS PAGE. Linker lengths (*L*) are shown above each lane. (b) Wild-type, full-length control (FLc) and arrested control constructs (Ac) for MATH *L*=55 resolved by SDS PAGE. In the full-length control the critical Pro at the end of the arrest peptide is mutated to Ala; in the arrested control a stop codon is inserted directly after the arrest-peptide. (c) Quantification of bands imaged from SDS PAGE for MATH wild-type *L*=29 showing fit of Gaussian distribution.

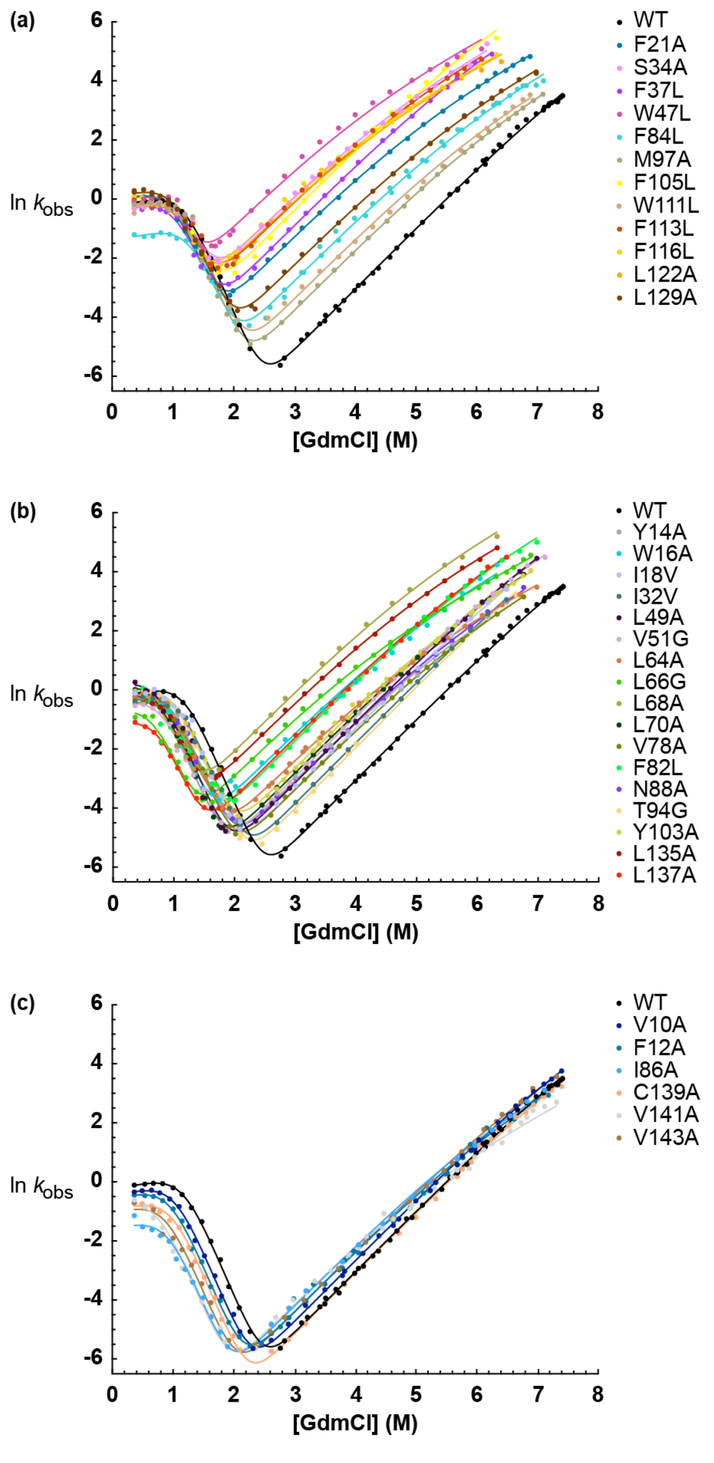

Fig. S2. Chevron plots of wild-type MATH and variants. A global fit of kinetic data reveals three classes of chevron: (a) low Φ-values, (b) medium Φ-values, (c) high Φ-values.

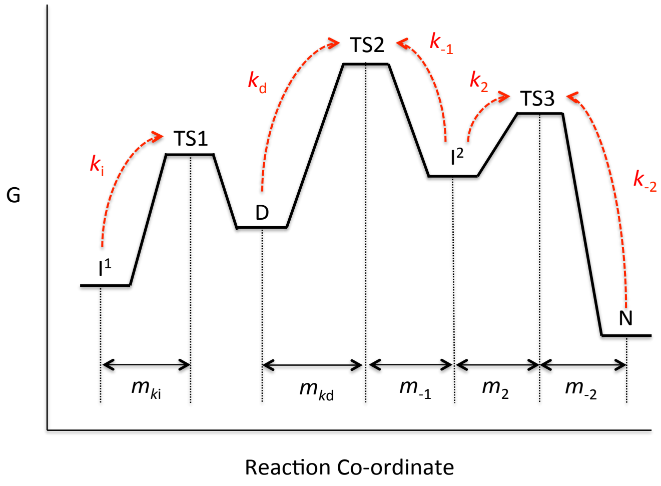

Fig. S3. Sequential transition state model adapted to include a low-energy folding intermediate, shown as off-pathway.

(a)

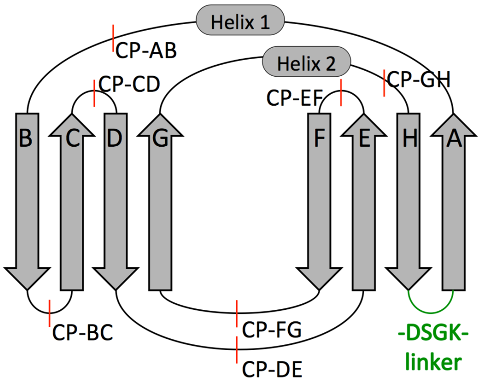

(b)

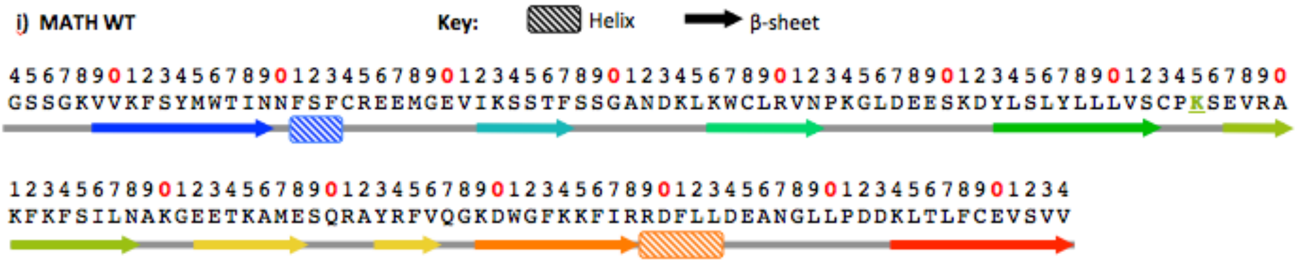

Fig. S4. Design of circular permutants. (a) Schematic view showing topology of MATH with positions of new termini in loop regions used to generate the circular permutants indicated (red bars). Original N and C termini are joined by a DSGK linker (shown in green) (See Materials and Methods). (b) MATH wild-type sequence with secondary structure assignment generated by the STRIDE algorithm (M. Heinig and D. Frishman, STRIDE: a web server for secondary structure assignment from known atomic coordinates of proteins, Nuc. Ac. Res. (2004) 32, 500-502). Secondary structure elements are coloured: strand A (blue), strand B (cyan), strand C (light green), strand D (dark green), strand E (olive), strand F (yellow), strand G (orange), strand H (red).

(a)

Wild-type

MRGSHHHHHHGLVPRGSGS

GSSGKVVKFSYMWTINNFSFCREEMGEVIKSSTFSSGANDKLKWCLRVNPKGLDEESKDYLSLYLLLVSCPKSEVRAKFKFSILNAKGEETKAMESQRAYRFVQGKDWGFKKFIRRDFLLDEANGLLPDDKLTLFCEVSVV

SGSGKFAYGIKDPIYQKTLVPGQQNATWIVPPGQYFMMGDWMSSFSTPVWISQAQGIRAGPGSSDKQEGEWPTGLRLSRIGGIH**

CP-CD

MRGSHHHHHHGLVPRGSGS

GLDEESKDYLSLYLLLVSCPKSEVRAKFKFSILNAKGEETKAMESQRAYRFVQGKDWGFKKFIRRDFLLDEANGLLPDDKLTLFCEVSVVQDSGKVVKFSYMWTINNFSFCREEMGEVIKSSTFSSGANDKLKWCLRVNPK

SGSGKFAYGIKDPIYQKTLVPGQQNATWIVPPGQYFMMGDWMSSFSTPVWISQAQGIRAGPGSSDKQEGEWPTGLRLSRIGGIH**

CP-DE

MRGSHHHHHHGLVPRGSGS

KSEVRAKFKFSILNAKGEETKAMESQRAYRFVQGKDWGFKKFIRRDFLLDEANGLLPDDKLTLFCEVSVVQDSGKVVKFSYMWTINNFSFCREEMGEVIKSSTFSSGANDKLKWCLRVNPKGLDEESKDYLSLYLLLVSAP

SGSGKFAYGIKDPIYQKTLVPGQQNATWIVPPGQYFMMGDWMSSFSTPVWISQAQGIRAGPGSSDKQEGEWPTGLRLSRIGGIH**

(b)

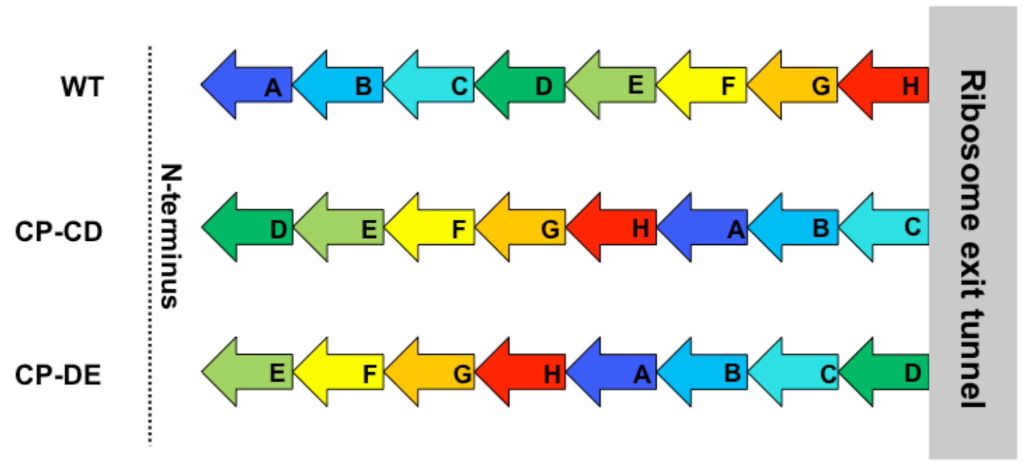

Figure S5 Wild-type and circular permutants of MATH in the arrest peptide assay. (a) sequences at L = 61. Blue, leader sequence; black, protein sequence (note all domains are same length); red, variable linker at L=61, Sec M sequence is underlined; Green, Lep domain; *, stop codon. (b) Schematic diagram showing order of emergence of β-stands from ribosome tunnel.
